# Liver X receptor activation induces apoptosis of melanoma cell through caspase pathway

**DOI:** 10.1186/1475-2867-14-16

**Published:** 2014-02-25

**Authors:** Wenjun Zhang, Hua Jiang, Jianlin Zhang, Yinfan Zhang, Antang Liu, Yaozhong Zhao, Xiaohai Zhu, Zihao Lin, Xiangbin Yuan

**Affiliations:** 1 Department of Plastic Surgery, Changzheng Hospital, 18F, No. 415 Fengyang Road, Shanghai, China

**Keywords:** Liver X receptor, Melanoma, T0901317

## Abstract

Liver X receptors (LXRs) are nuclear receptors that function as ligand-activated transcription factors regulating lipid metabolism and inflammation. Recent discoveries found LXRs could regulate tumor growth in a variety of cancer cell lines. In this study, we investigated the effect of LXR activation on melanoma cell proliferation and apoptosis both *in vitro* and *in vivo*. Treatment of B16F10 and A-375 melanoma cells with synthetic LXR agonist T0901317 significantly inhibited the proliferation of melanoma cells *in vitro*. Meanwhile, T0901317 induced the apoptosis of B16F10 melanoma cells in a dose-dependent manner. Furthermore, western blot assay showed that the pro-apoptotic effect of T0901317 on B16F10 melanoma cells was mediated through caspase-3 pathway. Oral administration of T0901317 inhibited the growth of B16F10 melanoma in C56BL/6 mice. Altogether, this study demonstrates the critical role of LXRs in the regulation of melanoma growth and presents the LXR agonist T0901317 as a potential anti-melanoma agent.

## Introduction

Melanoma is one of the most notoriously aggressive and treatment-resistant of human cancers, causing the majority (75%) of deaths related to skin cancers [1]. Although surgical excision remains to be a standard treatment at the early stages of the disease, it is ineffective after metastasis and patients with advanced disease have a poor prognosis [2]. Novel anti-cancer drugs and therapies are urgently needed to prevent and treat melanoma.

Liver X receptors (LXRα/NR1H3 and LXRβ/NR1H2) are nuclear receptors that could be activated by natural ligands like oxysterols, as well as synthetic agonists such as T0901317 [3]. High levels of LXRα expression can be found in liver, kidney, intestine, fat tissue and macrophages, while expressions of LXRβ are ubiquitous [4]. LXR agonists have been shown to be effective in treating cardiovascular diseases, metabolic syndromes, and murine models of atherosclerosis, diabetes and Alzheimer’s disease [5-8]. Recently, studies also show that LXR agonists exhibit anti-cancer activities in a variety of cancer cell lines. For example, LXR agonist T0901317 suppressed the proliferation of prostate, breast, colon, ovarian and leukemia cancer cells *in vitro*[9], and oral administration of T0901317 inhibited the growth of LNCaP tumors in C56BL/6 mice [10]. However, whether LXR agonist treatment could inhibit melanoma cell proliferation both *in vitro* and *in vivo*, and the underlying mechanisms, remain elusive.

Based on the previously reported anti-cancer activities of LXR agonists in multiple cancer cell lines, in this study, we sought to explore the effect of LXR agonist on B16F10 and A-375 melanoma cells and determine whether LXRs can be developed as a potential target in melanoma therapy.

## Materials and methods

### Reagents

Synthetic LXR agonist T0901317 was purchased from Cayman Chemical (Cayman Chemical, Ann Arbor, MI, USA). T0901317 used in the cell culture were diluted in DMSO (Sigma, Saint Louis, MO, USA). For xenograft model experiment, T0901317 was diluted in sesame oil (Sigma, Saint Louis, MO, USA) as previously reported [10]. caspase-3, cleaved caspase-3, actin antibodies were purchased from Cell Signaling Technology (Beverly, MA, USA).

### Cell culture

B16F10 murine melanoma cell line, A-375 human melanoma cell line and murine melanocyte cell line L10BIOBR were obtained from Cell Bank, Shanghai Institute of Life Science, Chinese Academy of Sciences (Shanghai, China). Cell lines were grown and maintained in DMEM supplemented with 10% fetal bovine serum (FBS) and antibiotics (100 U/mL penicillin and 10 μg/mL streptomycin) in a humidified incubator with 5% carbon dioxide, at 37°C.

#### Transfection of siRNA

The siRNA oligonucleotides against mouse LXRα, LXRβ, and control were purchased from Santa Cruz Biotechnology. B16F10 cells were transfected with siRNA oligonucleotides using GenJet transfection reagent (SignaGen, Gaithersburg, MD) according to the manufacturer’s instructions.

### Reverse transcription-PCR and quantitative PCR

Total RNA was isolated using Trizol reagent (Invitrogen) according to the manufacturer’s instructions. cDNA was synthesized with PrimeScript RT Master Mix (TAKARA, Dalian, China) according to the manufacturer’s instructions. The quantitative PCR was performed using SYBR Green PCR mix (Roche, Mannheim, Germany) on an ABI Prism 7900HT (Applied Biosystems). Thermocycler conditions included 2-minute incubation at 50°C, then 95°C for 10 minutes; this was followed by a 2-step PCR program, as follows: 95°C for 15 seconds and 60°C for 60 seconds for 40 cycles. β-actin was used as an internal control to normalize for differences in the amount of total RNA in each sample. The primer sequences were as follows (in the 5′ to 3′ orientation): β-actin forward, TGTCCACCTTCCAGCAGATGT, β-actin reverse, AGCTCAGTAACAGTCCGCCTAGA; ABCA1 forward, TACAGAAGCCAAGAAGGACC, ABCA1 reverse, TCGTCAGAGGTTTAAGTGGG; SREBF1 forward, TGGCTTGGTGATGCTATGTT, SREBF1 reverse, GGTTATTACCTGGTGGAGGG. Relative levels of gene expression were determined using the ΔΔCt method relative to the internal control gene β-actin.

### Cell proliferation assay

B16F10 and A-375 melanoma cells and murine melanocyte cell line L10BIOBR were cultured in 96-well plate at an initial density of 2000 cells per well, in 100 μl of DMEM medium containing 1% FBS with the addition of DMSO or T0901317 (1, 3 and 10 μM). After 24, 48 and 72 hours of DMSO or T0901317 treatment, cell proliferation was determined using a WST-1 Kit (Roche, Mannheim, Germany).

### Cell apoptosis analysis

Murine B16F10 melanoma cells were cultured in DMEM medium containing 10% FBS, and treated with DMSO or T0901317 (3 μM) for 24, 48 and 72 hours. Then, cells were harvested by trypsinization, and stained with FITC Annexin V and propidium iodide (PI) (Invitrogen, Paisley, UK). The stained cells were analyzed immediately by flow cytometry using FACS Calibur (BD Biosciences) and FlowJo software.

### Western blot

Cells were lysed in RIPA buffer (Beyotime, Haimen, China) and protein concentration was determined with the Bradford reagent (Beyotime, Haimen, China) using a bovine serum albumin standard. Proteins were resolved on 6% polyacrylamide gels containing SDS. Electrophoresis and blotting were performed using standard procedures. Visualization of the immunoreactive proteins was accomplished with an enhanced chemiluminescence reaction (Millipore, Billerica, MA, USA). Measurement of actin expression was used as a loading control.

### Mouse xenograft model

Six- to eight-week-old male C57BL/6 mice were subcutaneously injected with B16F10 melanoma cells at the dose of 10^6^ cells (n = 5 mice per group). Tumor growths were monitored by caliper measuring every other day, and tumor volumes were calculated using the formula: length × (width)^2^ × 0.52. The liver X receptor agonist T0901317 was administered by daily oral gavage in a sesame oil vehicle as previously reported [10]. All the mice were sacrificed and tumors were harvested for further analysis at indicated time points. Mice were treated and maintained at the Center for Laboratory Animals, Second Military Medical University.

### Statistical analysis

All experiments were repeated at least three times. Data were expressed as mean ± SEM. Statistical significance was determined by two-way analysis of variance followed by Student t test. Values of *p* less than 0.05 were considered significant.

## Results

### LXR expression in murine melanoma cell line

**Figure 1:**
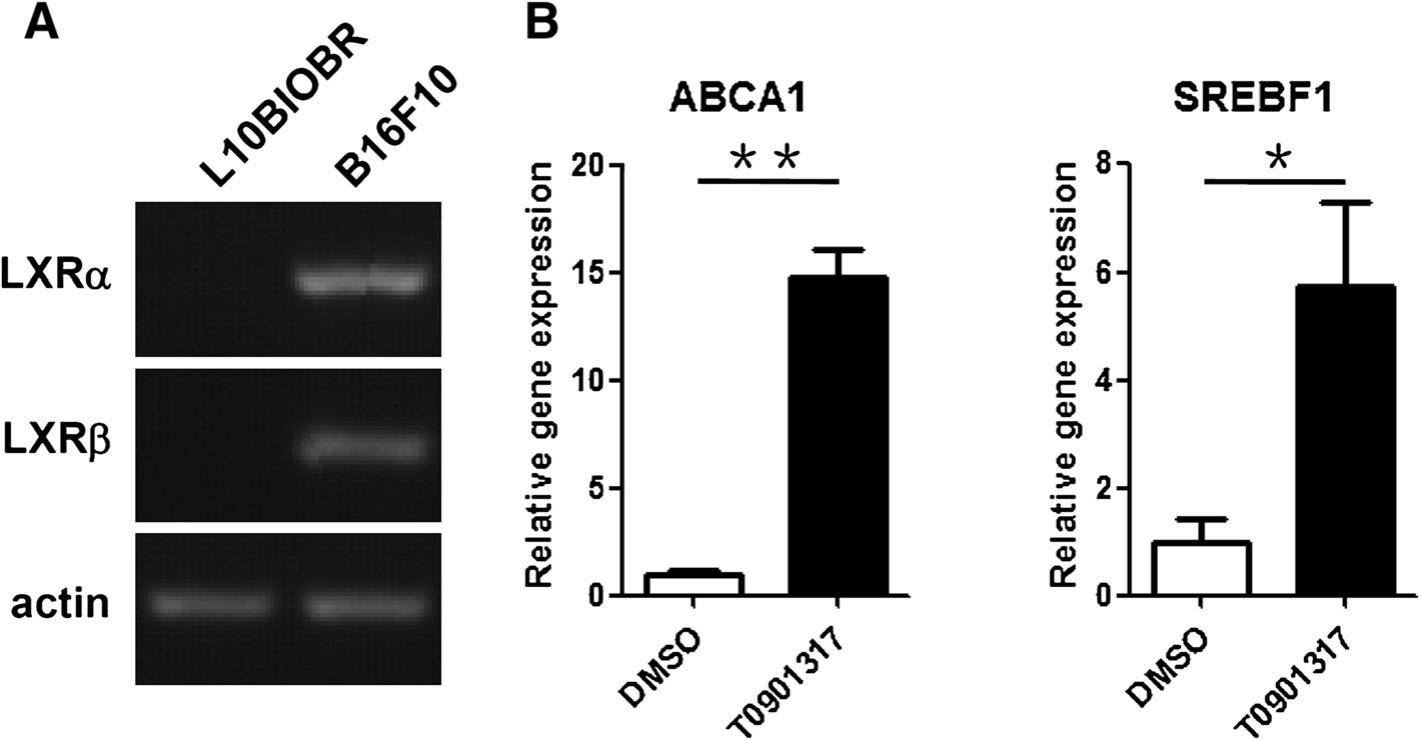
**Expressions of LXRs in B16F10 melanoma cells. (A)** Reverse transcription-PCR analysis of LXRα and LXRβ expression in B16F10 melanoma cells and melanocyte cell line L10BIOBR. **(B)** Real-time PCR analysis of expressions of LXR target genes (ABCA1 and SREBF1) in B16F10 melanoma cells upon T0901317 treatment. B16F10 melanoma cells were treated with DMSO or T0901317 (10 μM) for 48 hours. ^*^P < 0.05, ^**^P < 0.01.

To study the role of LXRs in melanoma development, we first investigated the expressions of LXRs (LXRα and LXRβ) in B16F10 murine melanoma cell line and murine melanocyte cell line L10BIOBR using reverse transcription-PCR. As shown in Figure 1A, both LXRα and LXRβ were expressed in B16F10 cells. However, expressions of LXRα and LXRβ were not detected in L10BIOBR. Upon T0901317 treatment, expressions of LXR target genes, ABCA1 and SREBF1, were induced in B16F10 (Figure 1B), indicating LXRs were functional in B16F10 melanoma cell line.

### LXR activation inhibits proliferation of melanoma cells

**Figure 2:**
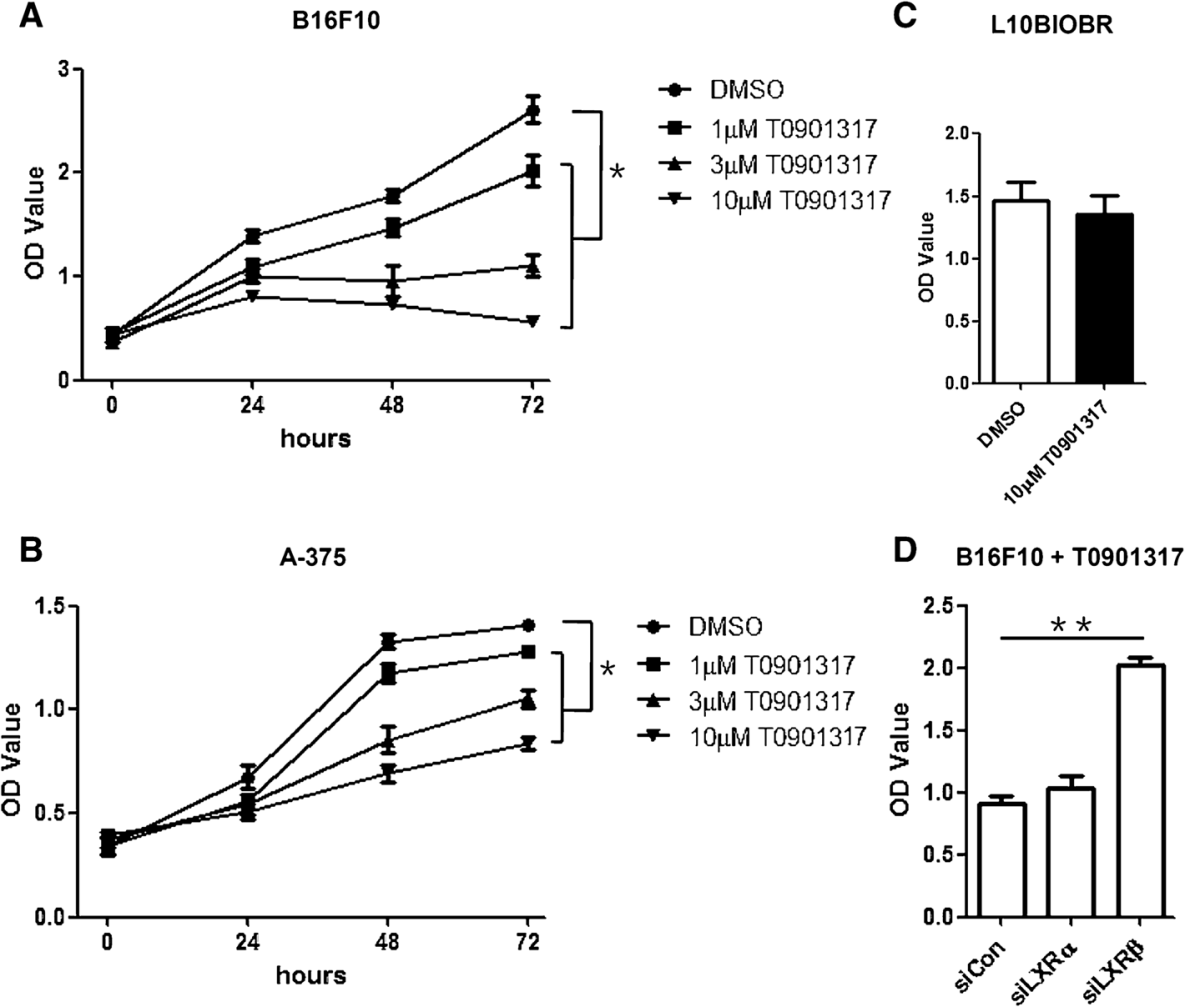
**LXR activation inhibits proliferation of melanoma cells. (A)** B16F10 melanoma cells were treated with increasing concentrations of T0901317 for 0, 24, 48 and 72 hours and tested for proliferation. **(B)** A-375 melanoma cells were treated with increasing concentrations of T0901317 for 0, 24, 48 and 72 hours and tested for proliferation. **(C)** L10BIOBR cells were treated with 10 μM T0901317 for 72 hours and tested for proliferation. **(D)** B16F10 cells were transfected with siRNA oligonucleotides. RT-PCR analysis was performed after 48 hours, and proliferation of B16F10 was measured after 72 hours in the presence of T0901317. ^*^P < 0.05.

As previously reported, LXR agonists are able to inhibit the proliferation of a variety of cancer cell lines. To determine the effect of LXR agonists on melanoma cell proliferation, we treated melanoma cell line B16F10 and A-375 with synthetic agonist T0901317. As determined by WST-1 assay, T0901317 suppressed the proliferation of B16F10 melanoma cell line in a dose- and time-dependent manner (Figure 2A, B). However, normal melanocyte proliferation was not affected by T0901317 (Figure 2C).

To test the specificity of LXRs in T0901317-mediated effects, we used small interfering RNAs (siRNAs) to down regulate the expression of LXRα and LXRβ in B16F10 cells. Although knockdown of LXRα did not reverse the anti-proliferative effects of T0901317, T0901317 failed to manifest the anti-proliferative effects in the absence of LXRβ (Figure 2D). Therefore, LXR activation by T0901317, expecially LXRβ, inhibits proliferation of melanoma cells.

### LXR activation induces apoptosis of melanoma cells via caspase pathway

**Figure 3:**
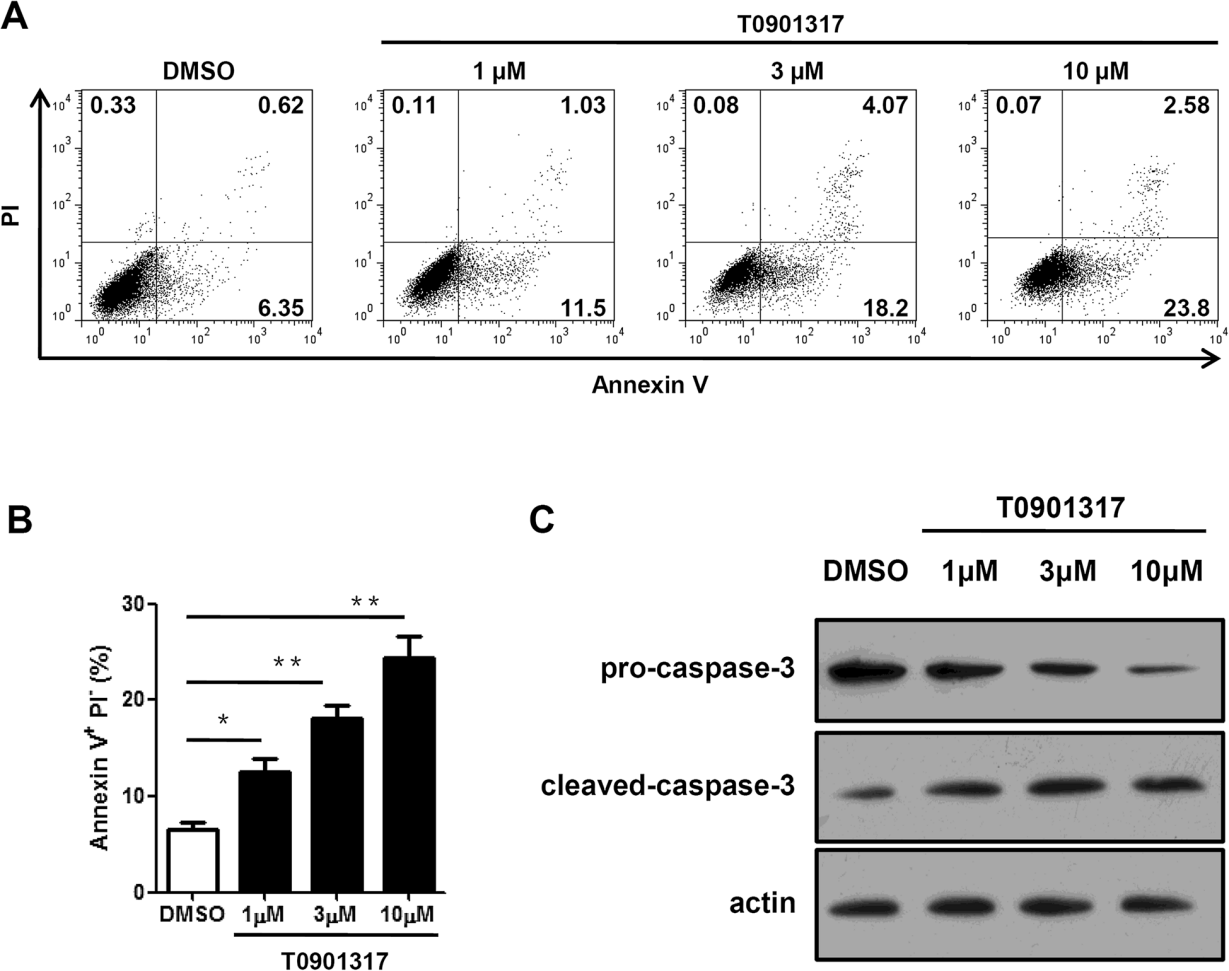
**LXR activation induces apoptosis of B16F10 melanoma cells via the caspase 3 pathway.** B16F10 melanoma cells were treated with DMSO or different dosage of T0901317 for 72 hours. **(A)** Cells were harvested and Annexin V/PI assays were performed to determine the effect of T0901317 on cell apoptosis by FACS. **(B)** Percentage of Annexin V^+^PI^-^ cells depicted in A was quantified. **(C)** Cells were harvested and expressions of caspase-3 were detected. Measurement of actin expression was used as a loading control. ^*^P < 0.05, ^**^P < 0.01.

Based on the anti-proliferation effects of LXR activation on melanoma cells, we then investigated the effect of LXR agonist T0901317 on melanoma cell apoptosis. B16F10 cells were treated for 72 hours with increasing doses of T0901317, and then collected for apoptosis assay. As shown in Figure 3A, compared with DMSO treatment, T0901317 treatment significantly increased the percentage of apoptotic Annexin V^+^ PI^−^ cells, with 10 μM T0901317 exhibiting the strongest pro-apoptotic effects.

To further understand the mechanisms by which LXR activation promotes B16F10 cell apoptosis, we examined the expressions of apoptotic pathway-associated proteins. As shown in Figure 3B, T0901317 treatment induced the activation of caspase-3, indicating caspase-3 was involved in the LXRs-regulated melanoma apoptosis.

### LXR activation inhibits tumor growth in mice

**Figure 4:**
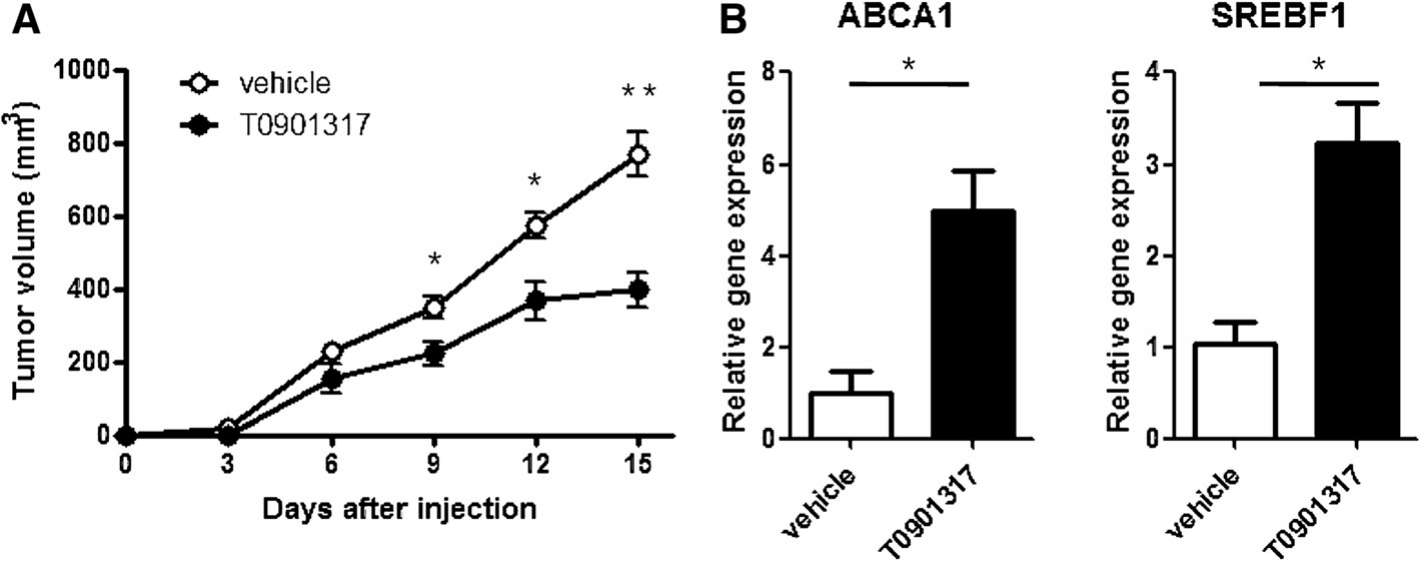
**Effect of T0901317 on melanoma growth in C56BL/6 mice. (A)** B16F10 melanoma cells (10^6^) were injected subcutaneously into flanks of the mice. The liver X receptor agonist T0901317 (10 mg/kg) or sesame oil vehicle was administered by daily oral gavage. Tumor growths were monitored every 3 days. **(B)** Expressions of LXRs target genes (ABCA1 and SREBF1) in tumor samples were analyzed by real-time PCR at day 15 when mice were sacrificed. ^*^P < 0.05, ^**^P < 0.01.

To confirm the anti-cancer effect of LXR agonist T0901317 *in vivo*, we established the murine melanoma xenograft model and treated mice with LXR agonist T0901317 simultaneously. T0901317 was administered via gavage using sesame oil vehicle at a dose of 10 mg/kg body weight per day as previously reported [10]. As shown in Figure 4A, the tumor volumes of T0901317-treated mice were significantly reduced when compared to vehicle-treated mice. To further confirm the *in vivo* anti-melanoma activity of T0901317 was mediated through LXR signaling, we analyzed the expression of LXR target genes, ABCA1 and SREBF1, in tumors, and found that expressions of ABCA1 and SREBF1 were increased in T0901317-treated melanoma (Figure 4B).

## Discussion

In this report, we present evidence of an anti-tumor effect for LXRs in a melanoma cancer system. Coupled with others’ results in prostate cancer, breast cancer, ovarian cancer, leukemia, hepatoma, non-small lung cancer and colon cancer cells [4,9,11], these data suggest that LXRs could inhibit tumor growth in multiple cancer systems.

Malignant melanoma is the most aggressive of the cutaneous malignancies [12], as current therapeutic treatments are greatly inadequate. Presently, the only effective therapy is the wide surgical resection of localized melanoma in its primary stages [13] and it is usually resistant to cytotoxic chemotherapy. Therefore, investigating the mechanisms of melanoma development and exploring novel drugs to treat melanoma are becoming more and more urgent.

Nuclear receptors are master regulators of transcriptional programs that integrate the homeostatic control of almost all biological processes [14]. LXRs are nuclear receptors that regulate lipid metabolism and inflammation [15]. Recently, LXRs were found to regulate the cancer development, and LXR agonists were found to be able to suppress the proliferation of multiple types of cancer cells, such as prostate cancer, breast cancer, ovarian cancer, leukemia, hepatoma, non-small lung cancer, colon cancer cells and so on [9]. Though Chuu *et al.* found that LXR agonists implicated antiproliferative effect on human melanoma cell line MDA-MB-435 [9], the mechanisms were elusive and whether LXR agonists could inhibit melanoma growth in vivo remains unknown. In our study, we found that synthetic LXR agonist T0901317 also suppressed the proliferation of B16F10 and A-375 melanoma cells both *in vitro* and *in vivo*, suggesting the critical role of LXRs in controlling melanoma progression and presenting LXR agonist as a potential anti-melanoma agent in the future.

Besides the anti-melanoma effects of LXRs, others have also reported that LXRs might have important biological roles in the skin. For example, LXRs could induce lipid synthesis in sebocytes, and inhibit the expressions of cytokines and metalloproteinases in skin-photoaging models [16-18]. LXRs are upregulated in melanocytes from perilesional skin of vitiligo patients [19], and LXR activation could inhibit the melanogenesis in human primary melanocytes and murine B16 melanoma cells by downregulating melanogenic enzymes through Ras- and ERK-induced MITF degradation [20]. Our study presented LXRs as key target proteins for the treatment of melanoma, which does not conflict others’ finding, depending on the use of different agonist and dosages. Further study is needed to fully explore the role of LXRs in the skin, including lipid synthesis, skin photoaging, melanogenisis as well as melanoma growth.

Up to now, the exact mechanisms by which LXR exerts anti-tumor effects are not clear. However, according to a previous study on LXR, LXR might exert anti-tumor effects through downregulation of AKT phosphorylation and inducing the canonical apoptotic pathway in prostate cancer [11]. It is possible that, in our study, LXR might also exert anti-melanoma effects through AKT signalling. In our future study, we will try to explore the molecular mechanisms.

In this study, we demonstrated that LXR activation inhibited the proliferation, and induced the apoptosis of melanoma cells. We proposed that synthetic agonist of LXRs, T0901317, might be a potential agent in treating melanoma.

## Competing interest

The authors declare no conflict of interest.

## Authors’ contributions

WZ and HJ participated in the design of this study and wrote the manuscript. JZ, YZ, AL collected important background information. YZ and XZ participated in performing the in vitro studies. ZL finished the in vivo study. XY performed the statistical analysis. All authors read and approved the final manuscript.
